# Features of *SRSF2*-mutated patients with chronic myelomonocytic leukemia in a national (ABCMML) and international cohort (cBioPortal)

**DOI:** 10.1007/s10354-025-01091-x

**Published:** 2025-06-23

**Authors:** Marc Tölly, Klaus Geissler

**Affiliations:** Medical School, Sigmund Freud University, Sigmund Freud Platz 3, 1020 Vienna, Austria

**Keywords:** Chronic myelomonocytic leukemia, *SRSF2*, Austrian biodatabase for chronic myelomonocytic leukemia, cBioPortal, Mutations, Chronische myelomonozytäre Leukämie, *SRSF2*, „Austrian Biodatabase for chronic myelomonocytic leukemia“, cBioPortal, Mutationen

## Abstract

The big data derived from major international collaborations now make it possible to validate findings from traditional national patient cohorts in order to demonstrate consistency. In this study, we compared findings in *SRSF2*-mutated patients of the Austrian biodatabase for chronic myelomonocytic leukemia (ABCMML) with the CMML cohort documented in cBioPortal. It has been consistently shown in both CMML cohorts that *SRSF2* mutations are not associated with shorter overall survival and acute myeloid leukemia-free survival. Regarding phenotypic features, it was found that *SRSF2* mutations were significantly associated with higher hemoglobin values in the ABCMML and cBioPortal cohorts. The number of platelets was significantly lower in *SRSF2*-mutated patients in both CMML cohorts, but there were no differences regarding leukocytes and circulating blasts. Data validation using two independent CMML cohorts ensures the quality standards for clinical decision-making.

**Supplementary Information:**

The online version of this article (10.1007/s10354-025-01091-x) contains supplementary material, which is available to authorized users.

## Abstract (de)

Die Validierung nationaler Patientenkohorten zur Überprüfung der Datenkonsistenz wird durch umfangreiche Daten aus internationalen Kooperationen ermöglicht. In dieser Studie verglichen wir die Ergebnisse von Patienten mit *SRSF2*-Mutationen aus der nationalen „Austrian Biodatabase for chronic myelomonocytic leukemia“ (ABCMML) mit jenen von Patienten, die in der internationalen Plattform cBioPortal dokumentiert sind. Eine Konsistenz der Ergebnisse in beiden Patientenkohorten fand sich im Hinblick auf ein nicht unterschiedliches Gesamtüberleben und ein von akuter myeloischer Leukämie (AML) freies Überleben bei Patienten mit oder ohne *SRSF2*-Mutation. In Bezug auf den Phänotyp zeigte sich, dass sowohl in der ABCMML- als auch in der cBioPortal-Kohorte *SRSF2*-Mutationen mit höheren Hämoglobinwerten assoziiert waren. Die Thrombozytenwerte waren bei Patienten mit *SRSF2*-Mutationen in beiden Kohorten mit chronischer myelomonozytärer Leukämie (CMML) vermindert, während es bei Leukozyten und zirkulierende Blasten keine Unterschiede gab. Diese Validierung von Daten in 2 unabhängigen CMML-Kohorten gewährleistet die erforderlichen Qualitätsstandards für klinische Entscheidungsfindungen.

## Introduction

Chronic myelomonocytic leukemia (CMML) is a rare, phenotypically and genetically diverse hematologic cancer that affects the elderly and has an inherent risk of developing into secondary acute myeloid leukemia (AML). According to the FAB criteria, CMML was initially separated into two groups based on the presence of myeloproliferation: myeloproliferative disorder (MP-CMML; WBC count > 13 × 10^9^/L) and myelodysplastic syndrome (MD-CMML; WBC count < 13 × 10^9^/L) [1, 2]. Since CMML is characterized by features of both MDS and MPN, the World Health Organization (WHO) classification of 2002 assigned CMML to the mixed category (MDS/MPN) [3]. After the 2016 revision to the WHO classification of myeloid neoplasms and acute leukemia [4], updated diagnostic criteria for CMML were recently reported by two groups [5, 6]. Patients with CMML have highly variable outcomes, suggesting that several factors can determine the course of disease and the causes of death in these patients [7–13].

We recently reported on the Austrian biodatabase for CMML (ABCMML) [14]. The epidemiologic, hematologic, biochemical, clinical, immunophenotypic, cytogenetic, molecular, and biologic data of patients with CMML have been gathered from various Austrian centers for 40 years, and the ABCMML has been demonstrated to be a representative and practical source of real-world data for biomedical research.

Because of the molecular heterogeneity of CMML, it is critical to understand the meaning of molecular characteristics so that patients can be provided with the best possible care for their unique circumstances. The effects of molecular aberrations on the clinical outcome and phenotype of the disease have been examined in a few studies, but the majority of these studies’ conclusions were not confirmed by separate cohorts. However, a prognostic parameter should not enter clinical practice unless it has been demonstrated that it performs a useful role. External validation denotes evaluation of the performance of a prognostic parameter in a sample independent of the one used to develop the model [15].

Big data containing a huge number of datasets from international large consortium efforts are now available for many cancer entities, including CMML. The cBioPortal platform is such a collection of big data that aims to build a platform to support clinical decisions for personalized cancer treatment [16]. Moreover, due to the large number of well-characterized patients, it is a perfect source of data for the validation of findings from traditional, sometimes much smaller patient cohorts. Mutations in the *SRSF2* gene are the second most common molecular aberration in CMML, following after *TET2* mutations [7]. In the current study, we used data from CMML patients documented in cBioPortal to validate the features of *SRSF2*-mutated CMML patients who have been analyzed in the ABCMML.

## Patients and methods

### Patients

#### ABCMML analysis

The ABCMML can serve as a representative and practical real-world data source for biological research, as we have recently demonstrated [14]. Epidemiologic, hematologic, biochemical, clinical, immunophenotypic, cytogenetic, molecular, and biologic data of CMML patients from various sites were gathered retrospectively and included in this database. Leukemic transformation and CMML were diagnosed based on the WHO criteria [2–4]. Clinical and routine laboratory parameters were obtained from patient records. A detailed central manual retrospective chart review was carried out to ensure data quality before analysis of the data from institutions. Patients with CMML in transformation were not included in this study. In 327 patients, mutation data were available to analyze overall survival (OS), acute myeloid leukemia (AML)-free survival, and differences in phenotypic parameters between mutated and wildtype patients. This research was approved by the ethics committee of the City of Vienna on 10 June 2015 (ethics code: 15-059-VK).

#### cBioPortal analysis

We selected the myelodysplastic syndromes dataset from cBioPortal [16] containing 399 CMML patients with data including sex, age, white blood cell (WBC) count, platelets, hemoglobin (Hb), overall survival, AML-free survival, bone marrow (BM) blasts, circulating blasts, cytogenetics, and gene mutations (http://www.cbioportal.org) to analyze OS, AML-free survival, and differences in phenotypic parameters between mutated and nonmutated patients.

### Statistical analysis

The log-rank test was used to determine whether individual parameters were associated with OS. Overall survival was defined as the time from sampling to death (uncensored) or last follow-up (censored); AML-free survival was defined as the time from sampling to transformation into AML or death (uncensored) or last follow-up (censored). The chi-squared test was used to compare dichotomous variables between groups. When continuous variables were not normally distributed, two unmatched groups were compared using the Mann–Whitney U test. At *p* < 0.05, the results were deemed significant. SPSS v. 27 (IBM Corp., Armonk, NY, USA) was used for statistical analyses; two-sided *p*-values were reported. Mutations with a variant allele frequency (VAF) of at least 5% in the ABCMML database and at least 2% in the cBioPortal platform were regarded as positive.

## Results

### Characteristics of patients and prevalences of *SRSF2* mutations

The baseline characteristics of both CMML cohorts are shown in supplementary Tables 1 and 2. Analyzed were 327 patients in the ABCMML cohort and 399 patients in the cBioPortal cohort. As seen in other CMML series, there was a male predominance in both cohorts, and more than half of the patients were aged 70 years or older [14]. All characteristics except leukocytes were comparable between cohorts. The proportion of patients with leukocytes > 13 G/L was significantly higher in the ABCMML cohort as compared to the cBioPortal cohort (57% vs. 32%; *p* < 0.001). The median leukocyte counts were 14.1 vs. 9.2 G/L in these cohorts, respectively. Regarding clinical outcome, median survival was 29.0 months in the ABCMML cohort as compared to 31.6 months in the cBioPortal cohort. The prevalences of *SRSF2* mutations were 38.7% (122/315) in the ABCMML group and 36.3% (139/383) in the BIOPOPRTAL group.

**Table 1:** Phenotypic features of ABCMML patients including leukocytosis, anemia, thrombocytopenia, and circulating blasts stratified by the presence or absence of an *SRSF2* mutation

Parameter	With *SRSF2* mutation (%)	Without *SRSF2* mutation (%)	*P*-value
WBC ≥ 13 G/L	63/122 (52)	89/193 (46)	0.356
Hb < 10 g/dL	28/122 (23)	71/193 (37)	0.013
PLT < 100 G/L	64/122 (52)	72/194 (37)	0.010
PB blasts present	17/99 (17)	44/165 (27)	0.097

**Table 2:** Phenotypic features of cBioPortal patients including leukocytosis, anemia, thrombocytopenia, and circulating blasts stratified by the presence or absence of an *SRSF2* mutation

Parameter	With *SRSF2* mutation (%)	Without *SRSF2* mutation (%)	*P*-value
WBC ≥ 13 G/L	56/139 (40)	65/244 (27)	0.006
Hb < 10 g/dL	41/144 (28)	106/253 (42)	0.009
PLT < 100 G/L	65/142 (46)	90/250 (36)	0.068
PB blasts present	26/118 (22)	62/215 (29)	0.195

### Impact of *SRSF2* mutations on survival and AML-free survival

**Fig. 1:**
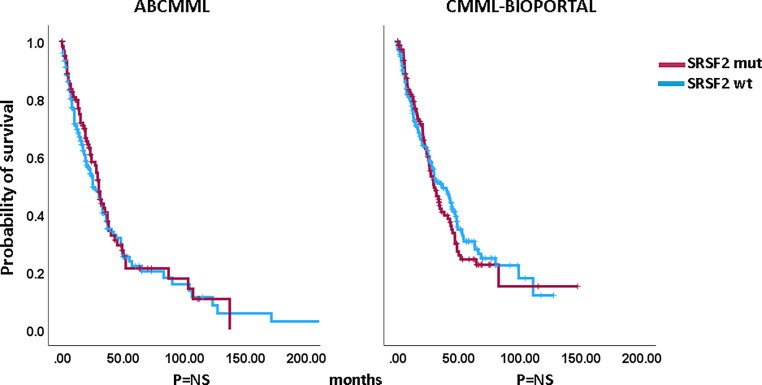
Kaplan–Meier plots for overall survival in CMML patients with and without *SRSF2* mutations

**Fig. 2:**
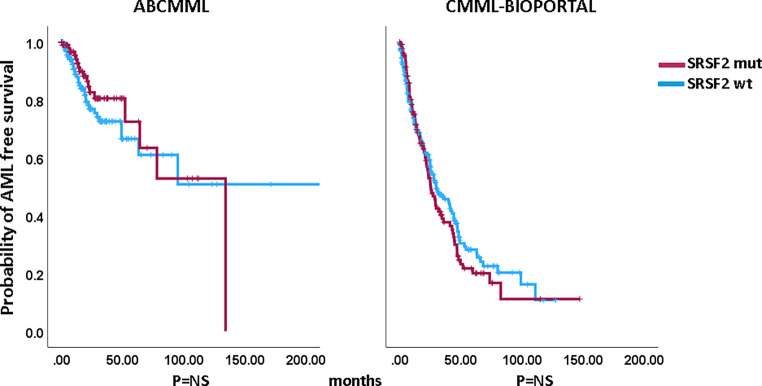
Kaplan–Meier plots for AML-free survival in CMML patients with and without *SRSF2* mutations

Figures 1 and 2 show the Kaplan–Meier curves of OS in *SRSF2*-mutated and *SRSF2*-nonmutated patients in both cohorts. There were no significant differences between mutated and nonmutated patients in either cohort. The median survival of *SRSF2*-mutated patients was 30.0 vs. 25.0 months (*p* = 0.588) among ABCMML patients and 29.7 vs. 36.3 months (*p* = 0.572) in the cBioPortal patients. Regarding AML-free survival, there was also no significant difference between *SRSF2*-mutated and *SRSF2*-nonmutated patients in either cohort: median AML-free survival was 134 months vs. not reached (*p* = 0.348) in the ABCMML cohort and 25.2 vs. 30.0 months (*p* = 0.418), respectively, in the cBioPortal cohort.

### Laboratory features in the presence or absence of *SRSF2* mutations

**Fig. 3:**
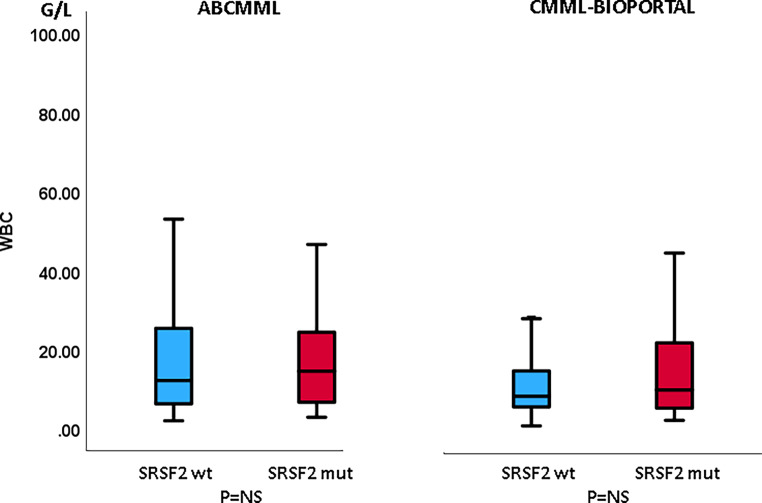
Boxplots showing the distribution of leukocytes in *SRSF2*-nonmutated and *SRSF2*-mutated CMML patients including median values, minimum values, maximum values, and upper and lower quartiles in both study cohorts

**Fig. 4:**
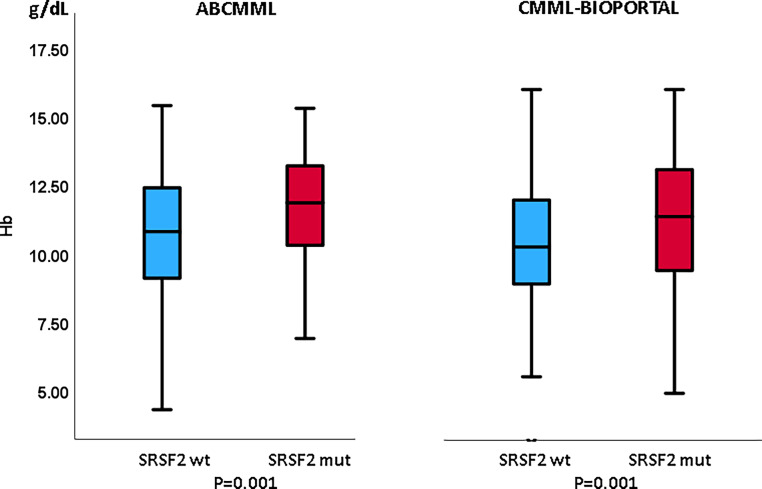
Boxplots showing the distribution of hemoglobin values in *SRSF2*-nonmutated and *SRSF2*-mutated CMML patients including median values, minimum values, maximum values, and upper and lower quartiles in both study cohorts

**Fig. 5:**
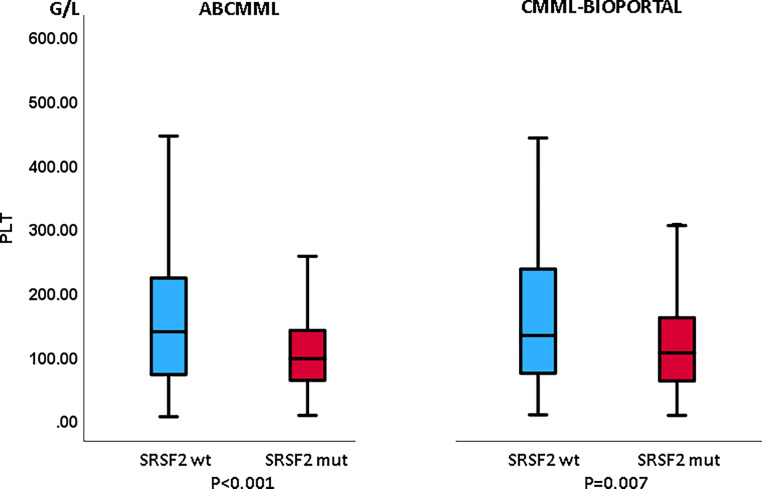
Boxplots showing the distribution of platelets in *SRSF2*-nonmutated and *SRSF2*-mutated CMML patients including median values, minimum values, maximum values, and upper and lower quartiles in both study cohorts

Tables 1 and 2 show the phenotypic parameters in the ABCMML and cBioPortal patients, respectively. The proportion of patients with leukocytosis > 13 G/L was significantly higher in *SRSF2*-mutated patients in the cBioPortal cohort but not in the ABCMML cohort. In both cohorts, the proportion of patients with anemia with Hb < 10 g/dl was significantly lower in *SRSF2-*mutated patients. The proportion of patients with thrombocytopenia < 100 G/L was significantly higher in *SRSF2*-mutated patients in the ABCMML cohort and the difference was of borderline significance in the cBioPortal cohort. The proportion of patients with circulating blasts was not different between mutated and nonmutated patients in either cohort. In Figs. 3, 4, and 5, metric values are visualized by boxplot diagrams. In both cohorts, the Hb values were significantly higher and the platelet values significantly lower in *SRSF2*-mutated patients as compared to wildtype patients. In the ABCMML cohort, the median values of *SRSF2*-mutated and nonmutated patients were 14.6 vs. 12.2 G/L for WBC, 11.9 vs. 10.8 g/dL for Hb, and 96 vs. 138 G/L for platelets, respectively. In the cBioPortal cohort, the median values of *SRSF2*-mutated and nonmutated patients were 10.6 vs. 9.0 G/L for WBC, 11.4 vs. 10.3 g/dL for Hb, and 105 vs. 132 G/L for platelets, respectively.

## Discussion

In this study we analyzed a national CMML cohort from Austria (ABCMML) and an international cohort of CMML patients (cBioPortal) regarding clinical, epidemiologic, and hematologic features in order to obtain information on the consistency and general validity of findings in *SRSF2*-mutated patients.

*SRSF2* is the most frequently mutated spliceosome gene in CMML, but neither it nor *SF3B1* or *U2AF35* mutations are prognostically relevant [17]. Regarding *SRSF2*, this was confirmed in our study: there was no impact of *SRSF2* mutation on OS or AML-free survival, neither in the ABCMML nor in the cBioPortal cohort.

Regarding the phenotype of CMML patients, however, there was an unexpected and consistent finding in both cohorts: in patients with an *SRSF2* mutation, the proportion of patients with anemia with Hb < 10 g/dL was lower than among wildtype patients. Moreover, the Hb values were significantly higher in patients with an *SRSF2* mutation than in patients without this mutation. The reason for this is unclear at the moment. Unfortunately, data on the administration of red blood cell transfusions are lacking in both cohorts; therefore, these data must be interpreted with caution.

Another consistent finding regarding the phenotype was a statistically significantly lower platelet count in *SRSF2*-mutated patients in both study cohorts. This observation was already reported by Itzykson in 2013 [18], and it thus confirms this finding. The impact of *SRSF2* mutation on the other blood parameters was inconsistent. Whereas the proportion of patients with leukocytosis was significantly higher in mutated patients than in wildtype patients in the cBioPortal cohort, this was not the case in the ABCMML cohort. Moreover, the number of leukocytes expressed in metric values did not differ, neither in the ABCMML cohort nor in the ABCMML cohort.

The main limitation of this study is its retrospective nature. Moreover, the proportion of patients with leukocytes > 13 G/L was significantly higher in the ABCMML cohort as compared to the cBioPortal cohort. The reason for this imbalance is not completely clear. Increased laboratory screening of asymptomatic persons in recent years may have detected some diseases, including CMML, in an earlier phase than in previous years. Therefore, older patient series may be enriched in patients with more advanced disease as compared to more recent series. In fact, we have seen a significant drop in the proportion of patients with MP-CMML from 66% to 48% since 2010 in the ABCMML (unpublished data).

Changes to the diagnostic criteria of CMML over time since its first description in 1982 represent another limitation of the ABCMML database, suggesting that this patient group is more heterogenous as compared to the cBioPortal group, which contains patients who were included over a shorter period of time. Furthermore, it needs to be considered that a proportion of patients in the ABCMML cohort, in particular older patients, did not consent to BM puncture. However, we do not believe that this greatly affected diagnostic accuracy, since persistent peripheral blood monocytosis is the most important diagnostic feature, and a genoclinical model has recently been described that uses mutational data, peripheral blood values, and clinical variables to predict the MDS vs. CMML diagnosis with high accuracy in the absence of a BM biopsy result [19]. Moreover, somatic mutations associated with CMML were not only detected in CMML patients confirmed by BM biopsy but also in 57% of patients with nondiagnostic BM features. Interestingly, the OS in mutated patients not confirmed by BM biopsy was indistinguishable from that of patients confirmed by BM biopsy, suggesting that the mutational spectrum is a much more sensitive parameter for the detection of myeloid malignancies as compared to BM morphology [20].

Recently, the disease-centered approach to healthcare administration has given way to a patient-centered approach [21]. The adoption of big data characterized by a large quantity of digital data that are continually generated by people within clinical care and everyday life will enable the implementation of personalized and precise medicine based on personalized information. Moreover, these data can be used for validation of findings from national cohorts and, thus, make an important contribution to improving patient management.

## Supplementary Information

Supplementary Table 1

Supplementary Table 2
